# β-galactosidase stability at high substrate concentrations

**DOI:** 10.1186/2193-1801-2-402

**Published:** 2013-08-27

**Authors:** Anja Warmerdam, Remko M Boom, Anja EM Janssen

**Affiliations:** Food Process Engineering Group, Wageningen University, PO Box 8129, Wageningen, EV 6700 The Netherlands

**Keywords:** β-galactosidase, *Bacillus circulans*, Galacto-oligosaccharides, Concentrated systems, Enzyme activity, Enzyme stability

## Abstract

Enzymatic synthesis of galacto-oligosaccharides is usually performed at high initial substrate concentrations since higher yields are obtained. We report here on the stability of β-galactosidase from *Bacillus circulans* at 25, 40, and 60°C in buffer, and in systems with initially 5.0 and 30% (w/w) lactose. In buffer, the half-life time was 220 h and 13 h at 25 and 40°C, respectively, whereas the enzyme was completely inactivated after two hours at 60°C. In systems with 5.0 and 30% (w/w) lactose, a mechanistic model was used to correct the *o* NPG converting activity for the presence of lactose, glucose, galactose, and oligosaccharides in the activity assay. Without correction, the stability at 5.0% (w/w) lactose was overestimated, while the stability at 30% (w/w) lactose was underestimated. The inactivation constant *k*_*d*_ was strongly dependent on temperature in buffer, whereas only a slight increase in *k*_*d*_ was found with temperature at high substrate concentrations. The enzyme stability was found to increase strongly with the initial substrate concentrations. The inactivation energy *E*_*a*_ appeared to be lower at high initial substrate concentrations.

**Electronic supplementary material:**

The online version of this article (doi:10.1186/2193-1801-2-402) contains supplementary material, which is available to authorized users.

## Introduction

For enzymatic production processes it is of interest to use highly concentrated conditions, since energy, water, and material costs can be saved. However, the activity and stability of enzymes are often investigated in aqueous systems, which may lead to irrelevant data. The enzyme activity of β-galactosidases, which is used in the production of galacto-oligosaccharides (GOS), in highly concentrated systems was studied before (Warmerdam et al. 2013a) and was found to be strongly influenced by the concentration of reactants and products. The high concentration of reactants and products may not only lead to more reactions taking place, but it will also lead to molecular crowding, which can have large effects on enzyme activity (Minton 2001; Ellis 2001).

Besides the enzyme activity, their stability can as well be strongly affected by molecular crowding (Minton 2001; Ellis 2001). In 1985, Arakawa and Timasheff (1985) have already described the stabilization of the protein structure of lysozyme in the presence of osmolytes. De Cordt et al. (1994) described the influence of high concentrations of polyalcohols and carbohydrates on the enzyme stability by substrate binding or preferential hydration. They observed various situations in which the presence of inert crowding agents increases the thermo-stability of proteins (Perham et al. 2007; Stagg et al. 2007; Zhou et al. 2008). Recently, Yadav (2013) described that the presence of sucrose and trehalose strongly increased the half-life time of α-amylase.

GOS are usually produced with β-galactosidase at high temperatures and at high substrate concentrations in industry. An advantage of reactions at high temperatures is the improved solubility of the substrates which makes higher substrate concentrations possible (Bruins et al. 2001). However, the inactivation of the enzyme is faster as well (Bruins et al. 2003).

The stability of β-galactosidase from *Bacillus circulans* was investigated before in systems with low lactose concentration or in absence of lactose (Mozaffar et al. 1984). Vetere and Paoletti (1998), and Song et al. (2011a) studied the stability of several isoforms of β-galactosidase from *Bacillus circulans* in aqueous systems. They found that the enzyme preparation was (partly) stable up to 50°C. The stability of free β-galactosidase from *Bacillus circulans* in systems with high lactose concentrations, which are usually used in production systems, has to our knowledge never been investigated before.

When using high initial substrate concentrations, it is important to investigate the effect of reactants in the activity assay. Baks et al. (2006) found that starch and its hydrolysis products may have large effects on the Ceralpha activity assay. This assay is comparable to the activity assay used for β-galactosidases with *o* NPG as an artificial substrate. Lactose and (some of) its conversion products are substrate for β-galactosidase as well as *o* NPG: they act as acceptor molecule for the enzyme-galactose complex, and they act as inhibitors and competitors (Warmerdam et al. 2013a; Borralho et al. 2002) (Warmerdam A, Zisopoulos FK, Boom RM, Janssen AEM: Kinetic characterization of β-galactosidases, submitted). In addition, galactose and glucose are usually found to be inhibitors for β-galactosidases (Warmerdam et al. 2013a; Greenberg and Mahoney 1982; Macfarlane et al. 2008; Prenosil et al. 1987) (Warmerdam A, Zisopoulos FK, Boom RM, Janssen AEM: Kinetic characterization of β-galactosidases, submitted). Because of the interactions of these carbohydrates, it is important to correct the *o* NPG activity measurements for their presence.

The aim of this study is therefore to investigate the stability of β-galactosidase from *Bacillus circulans* at various temperatures both in buffer, and in systems with initially 5.0 and 30% (w/w) lactose. The remaining enzyme activity is measured via the *o* NPG activity assay. The activity measurements are corrected for the effect of the carbohydrates present in the reaction mixture.

## Materials and methods

### Materials

Lactose monohydrate (Lactochem), Vivinal-GOS and a β-galactosidase from *Bacillus circulans* called Biolacta N5 (Daiwa Kasei K. K., Japan) were gifts from FrieslandCampina (Beilen, The Netherlands). Biolacta N5 was previously found to have a total protein content of 19 ± 3% (Warmerdam et al. 2013b). In all calculations, the total enzyme concentration was assumed to be equal to the total protein concentration, because the actual enzyme concentration is not known. Sulphuric acid, sodium hydroxide, *o*-nitrophenyl β-D-galactopyranoside (*o* NPG), *o*-nitrophenol (*o* NP), D(+)-glucose, D(+)-galactose, maltotriose, maltotetraose, maltopentaose, maltohexaose, and maltoheptaose were purchased from Sigma-Aldrich (Steinheim, Germany). Sodium carbonate, citric acid monohydrate, and disodium hydrogen phosphate were purchased from Merck (Darmstadt, Germany).

McIlvaine’s buffer was prepared by adding together 0.1 M citric acid and 0.2 M disodium hydrogen phosphate in the right ratio to achieve a pH of 6.0.

### Lactose conversion

The stability of Biolacta N5 was investigated in a 0, 5.0, and 30% (w/w) lactose-in-buffer solution in a temperature controlled batch reactor with an anchor stirrer at 150 rpm. A certain mass of lactose monohydrate and a certain mass of buffer were weighted, so that a final concentration of lactose was obtained on a weight basis of 5% and 30% (w/w). 30% (w/w) lactose is close to the solubility at 50°C. The lactose was dissolved at approximately 60°C prior to cooling the solution to the desired temperature. The initial reaction volume was 25 mL. Temperatures were kept at 25, 40, or 60°C. A volume of 1.0 mL of 2.0 g∙L^-1^ Biolacta N5 was added once the temperature was constant. Samples were taken at 30 s, 5, 10, 15, 30, 60, 120, 240, 360 minutes and 22, and 24 hours for determination of the carbohydrate composition (100 μL sample) and for determination of the enzyme activity (210 μL sample). The final reaction volume was 21 mL.

### Sample handling for determination of the carbohydrate composition

The sample (100 μL) taken from the reactor for determination of the carbohydrate composition was directly added into an Eppendorf tube with 50 μL of 5% (w/w) H_2_SO_4_ to inactivate the enzyme. Subsequently, the samples were stored at −20°C until further preparation.

Before HPLC analysis, the enzyme was removed from the samples by filtering the samples at 14,000 × g at 18°C for 30 minutes using pretreated Amicon® ultra-0.5 centrifugal filter devices (Millipore Corporation, Billerica, MA, United States) with a cut-off of 10 kDa in a Beckman Coulter Allegra X-22R centrifuge. The pretreatment of the filters consisted out of two centrifugation steps: first, 500 μL of Milli-Q water was centrifuged at 14,000 × g at 18°C for 15 minutes; and second, the filters were placed up-side-down in the tube and centrifuged at 14,000 × g at 18°C for 5 minutes. After filtration, the samples were neutralized with 5% (w/w) sodium hydroxide.

### Measurement of the carbohydrate composition

The filtered samples were analysed with HPLC using a Rezex RSO oligosaccharide column (Phenomenex, Amstelveen, the Netherlands) at 80°C. The column was eluted with Milli-Q water at a flow rate of 0.3 mL/min. The eluent was monitored with a refractive index detector.

The standards that were used for calibration of the column were lactose, glucose, galactose, maltotriose, maltotetraose, maltopentaose, maltohexaose, and maltoheptaose. Galacto-oligosaccharides up to a degree of polymerization of 7 were assumed to have the same response as the glucose-oligomers with an equal degree of polymerization. This was confirmed with mass balances.

### Enzyme activity measurements

The *o* NPG activity measurements, adapted from Nakanishi et al. (Nakanishi et al. 1983), were performed immediately after the sample was taken from the reaction mixture. An Eppendorf tube with 790 μL of 0.25% (w/w) *o* NPG-in-buffer was preheated in an Eppendorf Thermomixer at 40°C and 600 rpm for 10 minutes. Subsequently, 210 μL of sample was added and these mixtures were incubated for another 10 minutes at 40°C and 600 rpm. A volume of 1.0 mL of 10% (w/w) Na_2_CO_3_ solution was added to stop the reaction and, afterwards, the absorbance of *o* NP was measured at 420 nm. The *o* NP concentration was determined using the law of Lambert-Beer of which the extinction coefficient was determined to be 4576 M^-1^∙cm^-1^. The *o* NP formation was found to be linear during the first 10 minutes of the reaction. This initial rate of *o* NP formation was expressed in mmol∙min^-1^∙g protein^-1^. Measurements were performed in duplicate and the average enzyme activity was used.

### Modeling the effect of carbohydrates on the activity assay

The effect of carbohydrates on the *o* NPG activity assay can be described with a mechanistic model; we refer to previous work for the mechanistic description of the model (Warmerdam A, Zisopoulos FK, Boom RM, Janssen AEM: Kinetic characterization of β-galactosidases, submitted) (equation 1). This model accounts for the use of *o* NPG as substrate (*k*_1_) as well as acceptor (*k*_*a* 1_), the use of water as acceptor (*k*_*a* 2_), the use of lactose (lac) as substrate (*k*_3_) as well as acceptor (*k*_*a* 3_), the use of glucose (glu) as acceptor (*k*_*a* 4_), the use of galactose (gal) as acceptor (*k*_*a* 5_) as well as inhibitor (*K*_*i*_), and the use of oligosaccharides (oligo) as substrate (*k*_6_) as follows:

1 $$\frac{v_{0,\mathrm{oNP}}}{E_{0}}=\frac{k_{1}\left[\mathrm{oNPG}\right]}{1+\frac{k_{1}\left[\mathrm{oNPG}\right]+k_{3}\left[\mathrm{lac}\right]+k_{6}\left[\mathrm{oligo}\right]}{k_{a1}\left[\mathrm{oNPG}\right]+k_{a2}\left[H20\right]+k_{a3}\left[\mathrm{lac}\right]+k_{a4}\left[\mathrm{glu}\right]+k_{a5}\left[\mathrm{gal}\right]}+\frac{\left[\mathrm{gal}\right]}{K_{i}}}$$

where *v*_0, *oNP*_ is the initial rate of *o* NP formation in mM *o* NP∙s^-1^, *E*_0_ is the initial enzyme concentration in g protein∙L^-1^ or in mmol protein∙L^-1^ with the reaction rate constants *k*_*1*_, *k*_*a1*_, *k*_*a2*_, *k*_*3*_, *k*_*a3*_, *k*_*a4*_, *k*_*a5*_, and *k*_*6*_ in mmol *o* NP∙L∙(mmol X∙g protein∙s)^-1^ or in mmol *o* NP∙L∙(mmol X∙mmol protein∙s)^-1^, respectively, with X being the corresponding reactant. The inhibition constant *K*_*i*_ is in mM.

The respective parameters for Biolacta N5 were determined in previous work (Warmerdam A, Zisopoulos FK, Boom RM, Janssen AEM: Kinetic characterization of β-galactosidases, submitted) and are shown in Table 1.

**Table 1:** **Parameters for Biolacta N5 in the conversion of** ***o*** **NPG, lactose, glucose, galactose, and oligosaccharides**

*k*_1_ (mmol *o* NP∙L∙(mmol *o* NPG∙g protein∙s)^-1^)	0.10
*k*_3_ (mmol *o* NP∙L∙(mmol lactose∙g protein∙s)^-1^)	0.012
*k*_6_ (mmol *o* NP∙L∙(mmol oligos∙g protein∙s)^-1^)	0.077
*k*_*a* 1_ (mmol *o* NP∙L∙(mmol *o* NPG∙g protein∙s)^-1^)	0.0063
*k*_*a* 2_ (mmol *o* NP∙L∙(mmol H_2_O∙g protein∙s)^-1^)	0.0042
*k*_*a* 4_ (mmol *o* NP∙L∙(mmol glucose∙g protein∙s)^-1^)	0.00092
*k*_*a* 5_ (mmol *o* NP∙L∙(mmol galactose∙g protein∙s)^-1^)	0.023
*K*_*i*_ (mM)	255

To investigate the effect of the present reactants compared to when no reactants are added in the activity assay, we want to normalize this initial rate with the initial rate that would have been obtained without addition of carbohydrates, which is given by equation 2:

2 $$\frac{v_{0}{}_{,\mathrm{oNP}}}{v_{0,\mathrm{oNP}}^{\left[c\right]=0}}=\frac{1+\frac{k_{1}\left[\mathrm{oNPG}\right]}{k_{a1}\left[\mathrm{oNPG}\right]+k_{a2}\left[H20\right]}}{1+\frac{k_{1}\left[\mathrm{oNPG}\right]+k_{3}\left[\mathrm{lac}\right]+k_{6}\left[\mathrm{oligo}\right]}{k_{a1}\left[\mathrm{oNPG}\right]+K_{a2}\left[H20\right]+k_{a3}\left[\mathrm{lac}\right]+k_{a4}\left[\mathrm{glu}\right]+k_{a5}\left[\mathrm{gal}\right]}+\frac{\left[\mathrm{gal}\right]}{K_{i}}}$$

where $v_{0,\mathrm{oNP}}^{\left[c\right]=0}$ is the initial rate of *o* NP formation without addition of carbohydrates *C*. At each time point, the concentrations of reactants used in this equation is the concentration that has been measured with HPLC.

### Activity measurements corrected for the presence of carbohydrates

The activity measurements were corrected for the effect of lactose, glucose, galactose, and oligosaccharides on the activity assay with equation 3:

3 $$A_{\mathrm{corrected}}=\frac{A_{\mathrm{measured}}}{\frac{v_{0,\mathrm{oNP}}}{v_{0,\mathrm{oNP}}^{\left[c\right]=0}}}$$

where *A*_*measured*_ and *A*_*corrected*_ are the enzyme activity calculated directly from the absorbance measurements (see “Enzyme activity measurements”), and the enzyme activity corrected for the presence of lactose, glucose, galactose, and oligosaccharides, respectively.

For each sample made with Vivinal-GOS, the concentration of lactose, glucose, galactose, and total oligosaccharide was calculated. The concentrations of lactose, galactose, glucose and total oligosaccharides are 19 dm%, 1 dm%, 21 dm%, and 59 dm% in Vivinal-GOS. Oligosaccharides were assumed to be mainly trisaccharides with a molecular weight of 504 g/mol.

### Determination of enzyme stability

Enzyme inactivation during the running time of the experiment was modelled with a first order inactivation model with:

4 $$k_{t}=k_{0}\;\cdot \;e^{-k_{d}\cdot t}$$

where *k*_*0*_ and *k*_*t*_ are the reaction rates at time zero and time *t* in h, *k*_*d*_ is the enzyme inactivation constant in h^-1^, and *t* is the running time at which the sample was taken in hours. The enzyme inactivation constant *k*_*d*_ and the reaction rate at time zero *k*_0_ were determined by linearization of equation 4.

The inactivation energy *E*_*a*_ can be determined with the Arrhenius relation, equation 5:

5 $$k_{d}=k_{\infty }\;\cdot \;e^{-\frac{\mathrm{Ea}}{R\cdot T}}$$

where *k*_*d*_ and *k*_∞_ (the Arrhenius constant) are in s^-1^, *R* is the gas constant in J∙mol^-1^∙K^-1^, and *T* is the temperature in K.

The half-life time of the enzyme *t*_½_ can be determined with equation 6:

6 t12=1n2kd

## Results and discussion

### Effect of temperature and initial lactose concentration on enzyme stability

**Figure 1:**
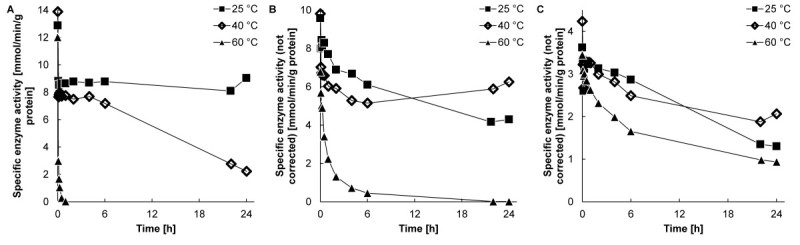
**Stability of Biolacta N5 at various substrate concentrations.** Residual enzyme activityof Biolacta N5 in **(A)** buffer, **(B)** 5.0% (w/w) lactose, **(C)** 30% (w/w) lactose at ■ 25, 40, xand ▲ 60°C and pH 6.0 with an enzyme concentration of 16 mg∙L^-1^. The enzyme activity is measured in the *o* NPG activity assay. (Lines for guidance).

Figure 1 shows the specific *o* NPG converting activity of Biolacta N5 after incubation in buffer (A), in 5.0% (w/w) lactose (B), and in 30% (w/w) lactose (C) at 25, 40, and 60°C. The observed data are not corrected yet for the presence of lactose, glucose, galactose, and Vivinal-GOS.

The initial activity in buffer was approximately 13 mmol∙min^-1^∙g enzyme^-1^, while the initial activities were approximately 10 and 4 mmol∙min^-1^∙g enzyme^-1^ in 5.0 and 30% (w/w) lactose, respectively. The reduction in the initial activity with an increasing lactose concentration is caused by the competition of lactose (that is present in the samples) with *o* NPG in the activity assay, as will be discussed later.

In buffer, the enzyme was stable at 25°C, but lost 84% of its activity after 24 hours of incubation at 40°C, and was completely inactivated after two hours at 60°C. This complete inactivation in buffer at 60°C was expected: Mozaffar et al. (1984), Vetere and Paoletti (1998), and Song et al. (2011b) described that its isoforms are stable up to at most 50°C for one hour. The stability at elevated temperatures improved considerably in the presence of lactose. In a 5.0% (w/w) lactose solution, it took six hours of incubation at 60°C before most of the activity was lost, while in a 30% (w/w) lactose solution, 27% of the enzyme activity was left after 24 hours at 60°C.

The measured activity in Figure 1B and C after 24 hours of reaction at 25°C was lower than at 40°C. One would expect a better stability at a lower temperature. These unexpected stability values are the result of the presence of reactants during the activity assay. These reactants interfere with the activity measurements similarly as was described by (Baks et al. 2006) (Warmerdam A, Zisopoulos FK, Boom RM, Janssen AEM: Kinetic characterization of β-galactosidases, submitted). Therefore, the carbohydrate composition in the samples was determined and the effect of these reactants on the activity assay was determined with equation 2.

### Carbohydrate profiles

**Figure 2:**
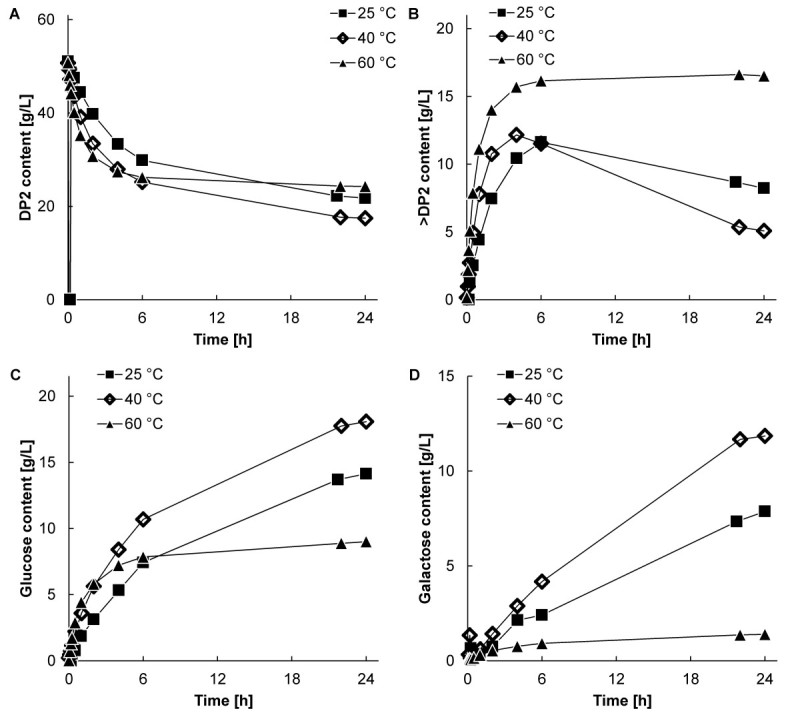
**Carbohydrate profiles at an initial lactose concentration of 5.0% (w/w) at ■ 25, 40, and ▲ 60°C and pH 6.0 with an enzyme concentration of 16 mg∙L**^**-1**^**. A**. Disaccharide conversion; **B**. GOS (all oligosaccharides larger than DP2) production; **C**. Glucose production; **D**. Galactose production. Figure corresponds with Figure 1B.

**Figure 3:**
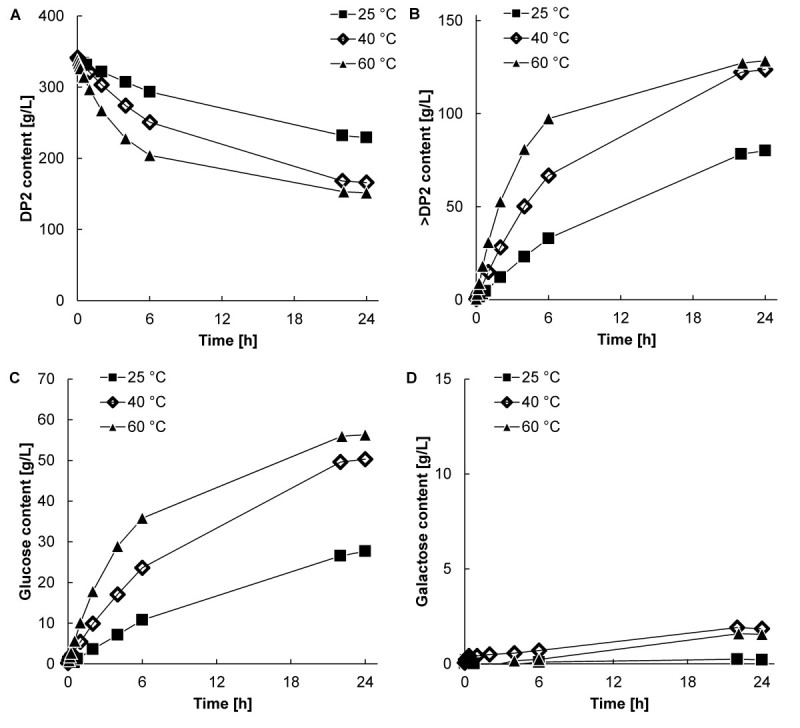
**Carbohydrate profiles at an initial lactose concentration of 30% (w/w) at ■ 25, 40, and ▲ 60°C and pH 6.0 with an enzyme concentration of 16 mg∙L**^**-1**^**. A**. Disaccharide conversion; **B**. GOS (all oligosaccharides larger than DP2) production; **C**. Glucose production; **D**. Galactose production. Figure corresponds with Figure 1C.

During the stability experiments described in Figure 1B and C, also samples were taken to determine the sugar composition. The results are presented in Figures 2 and 3. The concentrations of disaccharide (A), GOS (B), glucose (C) and galactose (D) are shown.

The carbohydrate content changed in time, and varied considerably between the initially different lactose concentrations. At an initial lactose concentration of 5.0% (w/w), the carbohydrate concentrations hardly changed anymore after 6 hours of reaction at 60°C. At 25 and 40°C the GOS content showed an optimum around 6 hours of incubation, indicating hydrolysis of the desired product at longer incubation times. Also a considerable amount of galactose was present after 24 hours of reaction. At an initial lactose concentration of 30% (w/w), GOS synthesis continued at all temperatures, including 60°C, until at least 22 hours of reaction (Figure 3B). Only small amounts of galactose were formed at all temperatures. The galactose production (indicating hydrolysis) was substantial at an initial lactose concentration of 5.0% (w/w) because of a high availability of water molecules, whereas no significant amounts of galactose were observed at an initial lactose concentration of 30% (w/w). It is clear that both the initial lactose concentration as well as the reaction temperature had a strong effect on the carbohydrate composition.

### Correction for the presence of carbohydrates in stability experiments

**Figure 4:**
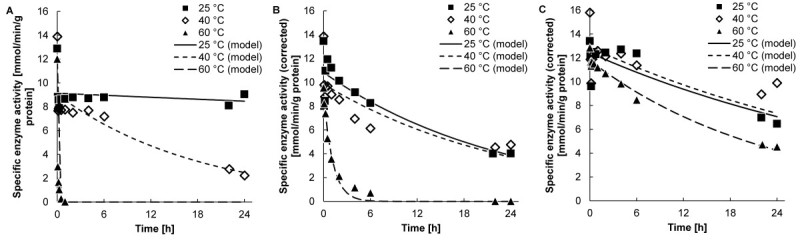
**Corrected stability of Biolacta N5 at various substrate concentrations.** Residual enzyme activity of Biolacta N5 in **(A)** buffer, **(B)** 5.0% (w/w), and **(C)** 30% (w/w) lactose at ■, ^______^25; ◇, - - - 40; and ▲, - - 60°C and pH 6.0 with an enzyme concentration of 16 mg∙L^-1^, corrected for the influence of lactose, galactose, glucose, and oligosaccharides. Symbols represent measured data, (dashed) lines represent modeled data.

The specific enzyme activity that was shown in Figure 1B and C was evaluated once more. The influence of lactose, galactose, glucose, and oligosaccharides on the *o* NPG activity assay was taken into account using equations 2 and 3. The carbohydrate content differed considerably during lactose conversion at various conditions (Figures 2 and 3) and the carbohydrates have a strong effect on the *o* NPG activity assay (Warmerdam et al. 2013a) (Warmerdam A, Zisopoulos FK, Boom RM, Janssen AEM: Kinetic characterization of β-galactosidases, submitted). The corrected enzyme activities are shown in Figure 4.

After correction for the presence of carbohydrates in the *o* NPG activity assay, the values of the specific enzyme activity on the Y-axis of Figure 4 are different. The enzyme activities at time zero are more or less similar (Table 2). The amount of added enzyme was the same in all experiments, thus a similar enzyme activity was indeed expected. Another aspect is the increase in the activity at 40°C in a 5.0% (w/w) lactose solution at longer incubation times. After correction this increase in activity is not present anymore and the activity decreased in time. After correction the activity decrease versus time at 25 and 40°C is more or less similar.

**Table 2:** **The initial** ***o*** **NP formation rate** ***k***_**0**_ **of Biolacta N5 in mmol∙min** ^**-1**^ **∙g protein** ^**-1**^ **at various initial lactose concentrations and temperatures, together with its 95% confidence interval**

[lactose]▶ ▼T	0% (w/w)	5.0% (w/w)	30% (w/w)
25°C	9.1 ± 1.0	11 ± 1	12 ± 1
40°C	9.0 ± 1.4	9.9 ± 1.2	13 ± 1
60°C	12 ± 1	8.9 ± 0.6	12 ± 0

The corrected data were used to fit the first order inactivation model (equation 4). The best fit is shown in Figure 4. The inactivation constant and the half-life time are shown in Tables 3 and 4. In buffer the half-life time at 25°C is about 4600 times higher as compared to the half-life time at 60°C. In 5.0% (w/w) lactose this value is only twenty times higher, while at 30% (w/w) lactose there is only a factor of two difference in the half-life time. At 25°C the enzyme appeared to be most stable in buffer, however, at 40 and 60°C the enzyme is most stable at elevated lactose concentrations. The half-life time of the enzyme (Table 4) (strongly) increased with increasing substrate concentration at 40 and 60°C.

**Table 3:** **The inactivation constant** ***k***_***d***_ **of Biolacta N5 in h** ^**-1**^ **at various initial lactose concentrations and temperatures, together with its 95% confidence interval**

[lactose]▶ ▼T	0% (w/w)	5.0% (w/w)	30% (w/w)
25°C	0.0032 ± 0.0112	0.043 ± 0.023	0.024 ± 0.011
40°C	0.054 ± 0.040	0.041 ± 0.025	0.024 ± 0.015
60°C	15 ± 3	0.85 ± 0.19	0.043 ± 0.006

**Table 4:** **The half-life time** ***t***_**½**_ **in h of Biolacta N5 in hours at various initial lactose concentrations and temperatures**

[lactose]▶ ▼T	0% (w/w)	5.0% (w/w)	30% (w/w)
25°C	220	16	29
40°C	13	17	29
60°C	0.048	0.82	16

**Figure 5:**
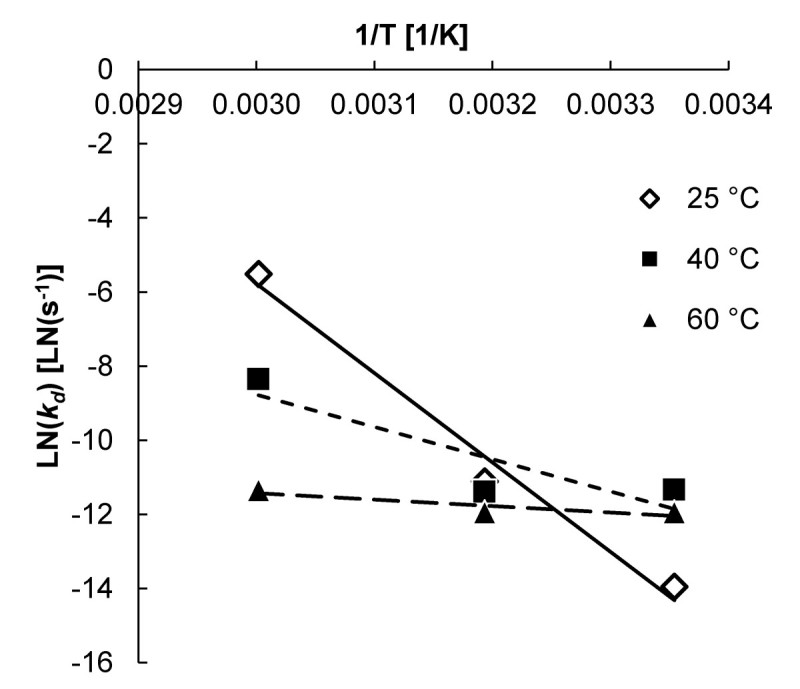
**Linearized Arrhenius plot of ln(*k***_***d***_**) as a function of 1/T.** Symbols: ◇, ^______^ 0% (w/w) lactose; ■, - - - 5.0% (w/w) lactose, and ▲, - - 30% (w/w) lactose.

Figure 5 shows the linearized Arrhenius plot of ln(*k*_*d*_) as a function of 1/T. The inactivation of the enzyme in buffer is strongly dependent on the temperature, whereas the inactivation in a system with 30% (w/w) lactose initially is hardly dependent on the temperature. The inactivation energy *E*_*a*_, shown in Table 5, decreased with increasing substrate concentration. This is similar to what was found by De Cordt et al. (1994). The higher stability of the enzyme is might be caused by molecular crowding or by complexation with the substrate or with a remaining galactose moiety.

**Table 5:** **Inactivation energy** ***E***_***a***_ **for various lactose concentrations**

[lactose]	***E***_***a***_
[% (w/w)]	[kJ∙mol^-1^]
0	200
5.0	72
30	14

A higher thermostability at high substrate concentrations is very favourable in the production of GOS by β-galactosidases from *B.circulans*. At high substrate concentrations, the reaction temperature can be higher than the enzyme’s stable ranges that were reported before in aqueous solutions, and it can be equal/closer to their optimal temperatures (Mozaffar et al. 1984; Vetere and Paoletti 1998; Song et al. 2011a), which will result in a higher enzyme stability.

## Conclusions

β-Galactosidase from *Bacillus circulans* was found to be quite stable against temperature at high substrate concentrations. For a proper conclusion on the remaining enzyme activity versus time it was important to correct the enzyme activity measurements for the presence of various reactants.

Without correcting the enzyme activity at 5.0% (w/w) lactose, the actual stability was overestimated, whereas not correcting the enzyme activity at 30% (w/w) lactose resulted in an underestimation of the actual stability of β-galactosidase from *Bacillus circulans*. A high initial lactose concentration had a large positive effect on the enzyme stability.

The improved stability in more concentrated systems is very interesting for production conditions. The utilization of more concentrated systems for enzymatic conversions is economically more interesting in order to avoid the unnecessary use of water, to save energy as a smaller volume needs to be heated, and to save on capital expenditures as less equipment is necessary.

## Authors’ original submitted files for images

Below are the links to the authors’ original submitted files for images. Authors’ original file for figure 1 Authors’ original file for figure 2 Authors’ original file for figure 3 Authors’ original file for figure 4 Authors’ original file for figure 5
